# Campaigning on the welfare state: The impact of gender and gender diversity

**DOI:** 10.1177/0958928716685687

**Published:** 2017-02-01

**Authors:** Laurenz Ennser-Jedenastik

**Affiliations:** University of Vienna, Austria

**Keywords:** Election campaigns, gender, gender diversity, social policy

## Abstract

Social policy matters have long been considered women’s issues. Extant research has documented a strong link between gender and the policies of the welfare state in the legislative, executive and electoral arenas. Yet what determines the strength of this association has largely been left unexplored. Drawing on tokenism theory, this article proposes gender diversity at the group level as a key explanatory factor. It hypothesizes that the gender gap in social policy diminishes as the female representation in a political party increases. To test this argument, it examines almost 8000 press releases issued by over 600 politicians during four election campaigns in Austria between 2002 and 2013. The analysis demonstrates that women talk more about social policy issues during election campaigns than men, but that this emphasis gap disappears for parties with a more equal gender balance. These results have important implications for our understanding of the politics of gender and social policy.

## Introduction

Social policy issues have long been viewed as ‘feminine’ issues. Women are typically perceived as the more caring and compassionate gender, and hence, the policy areas at the heart of the welfare state have been linked with stereotypically female traits (Huddy and Terkildsen, 1993). These stereotypes have profound consequences for the functional division of labour in the political realm. Across all regions of the world, women politicians are more likely to occupy legislative and executive functions that deal with stereotypically feminine issues, such as welfare, social insurance, healthcare or family affairs, than those dealing with finance, economics, foreign policy or defence (Reynolds, 1999). What is more, studies of parliamentary and electoral politics also find that women members of parliament (MPs) are more likely to take the floor in debates on social policy issues, and that such issues have greater prominence in the campaigns of female candidates than in those run by their male counterparts (Bäck et al., 2014; Kahn, 1996).

While existing research thus shows that the association between social policy and women is pervasive, it has little on offer to clarify the circumstances that contribute to the strength of this relationship. One explanation that has been hinted at in the literature is gender diversity at the group level (Bratton, 2005; Krook and O’Brien, 2012). The underlying assumption here is that greater equality in numbers will lead to less stereotype-conforming behaviour and functional segregation (Kanter, 1977a). Drawing on tokenism theory (King et al., 2010; Yoder, 1991), this article hypothesizes that female politicians are less likely to focus on stereotypically female issues, such as social policy, as the gender balance in a party becomes more equal.

To test this argument, the analytical section examines almost 8000 press releases issued by almost 700 party candidates during four election campaigns in Austria between 2002 and 2013. The analysis shows that (1) even when taking functional segregation into account, women are more likely than men to address social policy questions in election campaigns and (2) this gender gap is much more pronounced in male-dominated parties and disappears as the gender balance in a party becomes more equal.

This article thus presents some of the most conclusive evidence to date that the identification of social policy issues as the domain of female politicians is strongly conditional on gender diversity at the group level. This finding adds to our understanding of the drivers of the politics of social policy and the welfare state.

## Theoretical framework and hypotheses

### Social policy as a ‘women’s issue’

There is ample research showing that men and women are perceived to possess different character traits, and are therefore confronted with very different social expectations (e.g. Eagly and Karau, 2002; Prentice and Carranza, 2002). Women are typically viewed as the more caring and compassionate gender, while men are seen as more strong-willed and decisive (Huddy and Terkildsen, 1993). These diverging attributions of character traits have been theorized to originate in a traditional division of labour between the genders that is a result of physical sex differences (e.g. reproductive activities or upper body strength) interacting with the social and economic environment (Eagly, 1987; Eagly et al., 2000).

Such gender stereotypes also influence voter evaluations of political actors. For instance, Kahn (1992) reports that voters perceive women candidates as more compassionate and honest than men. In the absence of more specific information, voters also judge female candidates to be more liberal than their male counterparts (Koch, 2000; McDermott, 1998). Similar results have been found by Brown et al. (1993).

Moreover, gender stereotypes shape people’s views about who is better able to deal with certain policy issues. Especially on issues related to the welfare state, female politicians are judged to be more competent than their male counterparts. For instance, Alexander and Andersen (1993) report that voters judge female candidates to be more qualified to deal with childcare, poverty, healthcare and education, whereas men are seen as more competent in dealing with taxes, defence, foreign affairs and agriculture. This chimes with research by Huddy and Terkildsen (1993) who find that women candidates are viewed as more competent on ‘compassion issues’, such as helping the aged and the poor, childcare and child welfare. Similarly, Sanbonmatsu (2002a) reports that women candidates are viewed as better able to handle social security, whereas men are preferred on foreign affairs and crime. Gender stereotypes thus do not only influence the character traits we ascribe to male and female politicians but they also shape our views about politicians’ issue competence.

To be sure, the bulk of evidence on gender typing of policy issues originates from research conducted in the United States during the 1990s. Since gender relations are contingent on the cultural and political context, those findings may not translate one to one to other settings. For instance, gender roles may have changed substantially over time (Davis and Greenstein, 2009). When considering Western Europe in particular, one factor that clearly differs is the strength of trade unions, who – together with Social Democratic parties – have been strong proponents of generous welfare states and thus acquired ownership of many social policy issues. Also, trade unions are typically male-dominated, thus possibly mitigating the correlation between gender and welfare state issues.

Yet a substantial body of research that spans countries all over the world shows that variations in competence ascriptions are mirrored by a stark functional gender segregation when it comes to policy specialization among political elites. Some of the strongest evidence comes from studies on the allocation of ministerial portfolios. Davis (1997) finds that women are much more likely to get appointed to ministries of social welfare, health, family and youth than to the finance, defence, justice or interior portfolios. These results have been reproduced in a number of studies. Reynolds (1999: 564) observes that the link between gender and social policy portfolios holds across continents: ‘Whether in Europe, Africa, or Asia, there remain only a handful of women across the continent in the four key ministries of Foreign Affairs, Finance, Home Affairs, and Defense’ (see also Siaroff, 2000: endnote 15). Borrelli (2002) reports very similar findings for the cabinets of US presidents, where women are often appointed as Secretaries of Health, Welfare or Education. Along the same lines, women ministers in Latin American cabinets are a relatively strong presence in portfolios dealing with health, welfare, education, the environment and family policy (Escobar-Lemmon and Taylor-Robinson, 2005).

The pervasiveness of gender segregation across policy areas also influences committee assignments in legislatures. Diaz (2005), Coffé and Schnellecke (2013), and Heath et al. (2005) show that women legislators are less underrepresented in social policy committees (health, welfare, social insurance and education) than in other areas. These skewed distributions are, however, not only present in national parliaments but also in local government (Yule, 2000), and appear to be driven by self-selection rather than open discrimination (Bækgaard and Kjaer, 2012; Thomas, 1994).

The association of social policy issues with women politicians also applies to public speaking. Bäck et al. (2014) examine 50,000 speeches made in the Swedish *Riksdag* between 2002 and 2010 and find that women MPs – who speak less often than men overall, despite the high levels of gender parity in the Swedish parliament – are considerably less likely to take the floor on issues, such as macroeconomics, finance or transportation. Yet the gender gap in speech-giving disappears for issues, such as health, welfare and education. Similarly, evidence on bill initiation in Latin America demonstrates that women legislators devote a greater share of their effort to promoting bills that deal with children and family issues, education and health policy and women’s issues (Schwindt-Bayer, 2006). These findings chime with many studies from the US Congress that find women legislators to be especially active in advancing policies dealing with social policy issues (Swers, 2002; Thomas, 1994; for an overview, see Wängnerud, 2009).

Yet the functional segregation along gender lines in politics is not confined to the legislative and governmental arenas. The literature on electoral campaigning has also reported significant differences in issue emphasis between men and women – a pattern that appears to follow an electoral rationale. As Herrnson et al. (2003) report, women candidates who emphasized stereotypically female issues and targeted women’s groups in their campaigns gained an electoral advantage from this strategy.

It may therefore be little surprise that a number of studies on US elections have identified a gender gap in campaign issue emphasis. The analyses by Kahn (1993, 1996) show that women candidates for the US Senate are much more likely than men to discuss social policy issues in their campaign ads. This result has been replicated in a study of candidates for the US House of Representatives (Dabelko and Herrnson, 1997), and also for sub-national races in the United States (Larson, 2001). In an analysis of four Nordic countries, Bjarnegård et al. (2015) demonstrate that this finding is not limited to the context of US congressional elections. However, it is worth noting that not all study designs yield significant gender differences (Dolan, 2005).^1^

In addition to the direct effects of gender on the saliency of social policy, there are also indirect effects. These indirect effects have been shown to work through parties and intra-party institutions. As Kittilson (2011) finds, a party will dedicate more space to social justice issues as its share of women MPs and women in the party executive increases. Also, the presence of a dedicated women’s organization within a party will result in a higher salience of welfare policy in the party’s election manifesto.

Taken together, it must be concluded that the evidence for the association of social policy issues with women politicians is overwhelming (even though the evidence from the campaigning literature is mostly derived from studies of the United States). It stretches from voter ascriptions of traits and issue competence to the division of labour in executives and legislatures, to issue strategies in election campaigns. The first hypothesis flows directly from this body of research.

*H1*. Women are more likely than men to emphasize social policy issues in election campaigns.

### Explaining the link between gender and social policy: the impact of gender composition

While the evidence for the gender gap regarding social policy is extremely strong, it is important not to overlook the ample variation in these male–female differences that remains to be explained.

One potential determinant that has been hinted at is gender composition at the group level – the underlying assumption being that greater inequality in quantitative terms will lead to qualitative changes in individual-level behaviour and thus produce outcomes that are more structured according to gender stereotypes.

Social psychologists and sociologists have termed this idea tokenism. Tokenism theory originates from the works of Kanter (1977a, 1977b) and Laws (1975). Kanter’s study showed that women in a heavily male-dominated work environment experienced heightened visibility, resulting in greater performance pressures. They also reported greater levels of social isolation, feeling that ‘their differences from male peers were exaggerated’ (Yoder, 1991: 179). Finally, the women in Kanter’s study also experienced role encapsulation, thus being forced into stereotypically female behaviours.

While Kanter attributed her results purely to the skewed numbers (thus arguing that men in female-dominated occupations would have similar experiences), this point was later challenged. In her review of research on tokenism, Yoder (1991) argues that, in addition to gender composition, gender status (men being the dominant gender), occupational inappropriateness (women working in stereotypically male professions) and intrusiveness (the increasing share of women in a profession) may account for Kanter’s findings.

A number of studies have found support for this conditional view (King et al., 2010; McDonald et al., 2004; Yoder, 1994). Women in male-dominated environments (but not men in female-dominated environments, hence the importance of gender status) experience greater visibility and isolation and feel pressure to conform to stereotypically female roles.

In political science, Kanter’s (1977a, 1977b) ideas have featured prominently under the label of critical mass theory (Dahlerup, 1988) – a theory that was sometimes misread to stipulate that a qualitative shift in women-friendly political behaviour will emerge after women pass a 30-percent representative threshold (Childs and Krook, 2008). While studies indicate that no such magical threshold exists (Grey, 2002; Studlar and McAllister, 2002; see also Paxton et al., 2007: 275–276), it still holds true that an enhanced presence of female politicians – no matter the exact proportions in a legislature – helps promote a women-friendly agenda (Bratton, 2005; Bratton and Ray, 2002; Taylor-Robinson and Heath, 2003; Thomas, 1994; Wängnerud, 2009).

Harking back to tokenism theory, it can be argued that the roles female politicians perform will vary with the gender balance in their environment. Since politics is still a male-dominated business in most places (meaning that gender status and occupational inappropriateness are important in addition to numbers), women politicians in male-dominated political groups can be hypothesized to face greater performance pressures, social isolation and role encapsulation. The tendency to self-select into political roles and policy specializations that conform to stereotypically female characteristics is thus a function of the gender composition at the group level.

Empirical evidence supports these assumptions. Krook and O’Brien (2012), for instance, show that the pattern of appointing female ministers to stereotypically feminine policy portfolios is much less pronounced as the proportion of women in parliament increases. In countries with a more substantial female presence in the legislature, it is more likely that women will be appointed to ministries dealing with other policy areas. Also, parties are more likely to select females for the leadership (a stereotypically male role) when they have a more equal gender balance among their MPs (O’Brien, 2015; Wauters and Pilet, 2015).^2^ Similarly, Bratton (2005) demonstrates that gender differences in legislative behaviour diminish as the share of female representatives in US state legislatures grows.

In the context of European parliamentary systems, the most important group of reference for individual politicians is, of course, their party. Parties and the institutional framework they create also play a major role in how likely women are to enter positions of power (Caul, 1999; Kunovich and Paxton, 2005; Sanbonmatsu, 2002b), for instance, by adopting gender quotas in candidate selection (Caul, 2001).

In combination, these arguments amount to the expectation that the gender gap in emphasizing social policy issues (*H1*) shrinks as parties’ gender diversity increases.

*H2.* The gender gap in the emphasis on social policy shrinks as the gender balance in a party becomes more equal.

## Case selection, data and method

To test the two hypotheses outlined above, 7850 press releases issued by 683 politicians in four parliamentary elections held in Austria between 2002 and 2013 are examined. Austria is a useful case since it has one of the most generous welfare states in Europe, built up after 1945 by the two dominant parties, the Social Democratic Party of Austria (SPÖ) and the Christian Democratic Austrian People’s Party (ÖVP). High union density and strong links between the major parties and corporatist organizations have produced a system of consociational politics that has made it extremely difficult to reduce the level of social provisions. As a result, up to this day the welfare state remains one of the most salient policy issues, for both parties and voters (Dolezal et al., 2014; Kleinen-von Königslöw et al., 2014).

What is more, Austrian parties are highly diverse in terms of gender composition (Eder and Müller, 2013; Steininger, 2000). The share of women MPs in the period of observation ranges from 10 percent (the Alliance for the Future of Austria, BZÖ, in 2008) to 59 percent (the Greens in 2002). The Greens and the SPÖ (and in 2015 also the ÖVP) have adopted gender quotas for candidate selection, whereas the populist radical right-wing Freedom Party (FPÖ) and BZÖ reject such quotas on principle, as does the populist Team Stronach.

The dataset includes all press releases issued by individual politicians during the last 6 weeks of each campaign. Press releases in Austria are distributed centrally through the national news agency, APA, and made available for free on a website (http://www.ots.at). This makes them an important messaging tool, since it gives journalists and the public easy and comprehensive access to parties’ campaign messages. However, this should in no way be taken to mean that parties can exert centralized control over who sends out which press release. While some coordination may certainly be going on, press releases are regularly issued by hundreds of politicians in different institutions (central office, government, parliament) at all levels, including from regional branches and ancillary organizations (e.g. trade union factions and social partnership organizations), thus capturing much of the diversity of the parties.

Even though the number of press releases produced by party actors during a campaign is too overwhelming to be fully covered by the media, it has repeatedly been demonstrated that press releases in Austria shape media content to a considerable degree (Haselmayer et al., 2015; Seethaler and Melischek, 2014). They thus represent a valid measurement for party issue salience during the campaign, and in contrast to manifestos, campaign posters or TV ads, they are not the sole domain of national party elites but also of backbench MPs, party policy specialists, leaders of ancillary organizations, mid- and low-level elites and regional officials, thus allowing for an analysis of a much broader set of party actors. In total, the dataset covers 683 different individuals issuing at least one press release across the four election campaigns.^3^

A number of trained student coders recorded for each press release the name of the sender (up to two individuals) and his or her party affiliation as well as the main policy issue addressed in the title and subtitle (and a host of other variables that are not relevant to the present purpose). The issue coding scheme is extremely fine-grained, providing more than 650 policy categories (for more details of the coding scheme, see Dolezal et al., 2016).^4^

The dependent variable for the analysis is ‘social policy emphasis’, a dichotomous indicator coded 1 if the issue recorded for title or subtitle addresses any of the following policy areas: poverty, support for the unemployed (though not unemployment as a macroeconomic phenomenon), care and healthcare, pensions, social housing and housing subsidies, family and child benefits and childcare, as well as generic references to the welfare state (see Table A3 in the online appendix for more details).

The main independent variables are the gender of the individuals issuing the press release,^5^ and the gender balance within each party, measured as the proportion of female MPs in the party’s parliamentary party group (PPG) right before each election, for which data are taken from recent work on gender voting in Austria (Eder and Müller, 2013).^6^

Table 1 presents descriptive statistics for these variables by party. The figures suggest that issue ownership – typically ascribed to Social Democrats, to some extent also to Christian Democrats (Blomqvist and Green-Pedersen, 2004; Schumacher et al., 2013) – is an important factor. Social policy is most salient for the SPÖ and to a lesser degree for the ÖVP, even less so for the three populist parties (FPÖ, BZÖ and Team Stronach), and least important for the Greens. By contrast, the share of female senders as well as the proportion of women in the PPG declines steadily as we move from the left (Greens, SPÖ) and the centre-right (ÖVP, Team Stronach) to the far right (FPÖ, BZÖ). The striking correlation of these two variables with party ideology will certainly have to be accounted for in the analysis.

**Table 1.** Proportion of social policy press releases and press releases with female senders by party.

Party	Press releases	% press releases on social policy	% press releases by female senders	Coverage	% women in PPG (average across observations)
SPÖ	3126	22	40	2002–2013	35
ÖVP	2113	18	26	2002–2013	27
FPÖ	1181	16	9	2002–2013	17
Greens	651	12	50	2002–2013	53
BZÖ	667	16	8	2006–2013	12
Team Stronach	112	16	23	2013	20
Total	7850	19	30		29

PPG: parliamentary party group.

The BZÖ was founded in 2005 and Team Stronach in 2012.

Aside from the explanatory variables operationalizing the two hypotheses, the statistical models include a number of control variables that may be linked to both gender and social policy emphasis. Central among them are functions in government and parliament, such as ministers and junior ministers holding portfolios dealing with the issues coded in the dependent variable. An indicator for ministers and junior ministers in the social affairs, health and family portfolios is therefore included. Another predictor denotes whether a politician was at the time a member of the parliamentary committees for social policy, health or families.

Furthermore, the models control for union officials (assuming that trade unionists talk more about social policy), elite status (members of government, speakers of parliament, party leaders, party floor leaders and party secretaries), government status and party ideology (using data from the Chapel Hill expert surveys, see Bakker et al., 2015). In addition, random effects at the party-election level capture unobserved heterogeneity between parties at each election (fixed effects versions of the models are reported in the online appendix). Table 2 presents descriptive statistics for all independent variables that will enter the analysis.

**Table 2.** Descriptive statistics for independent variables.

Variable	N	Mean	SD	Min	Max
Female sender	7850	0.30	0.46	0	1
Share of women in PPG	7850	29.32	10.77	9.50	58.80
Minister (social policy portfolio)	7850	0.01	0.10	0	1
Member of social policy committee	7850	0.15	0.36	0	1
Trade union official	7850	0.04	0.20	0	1
Government party	7850	0.47	0.50	0	1
Elite politician	7850	0.34	0.48	0	1
Party ideology (0–10)	7850	5.64	2.31	2.17	9.67

SD: standard deviation; PPG: parliamentary party group.

## Analysis

**Figure 1.**
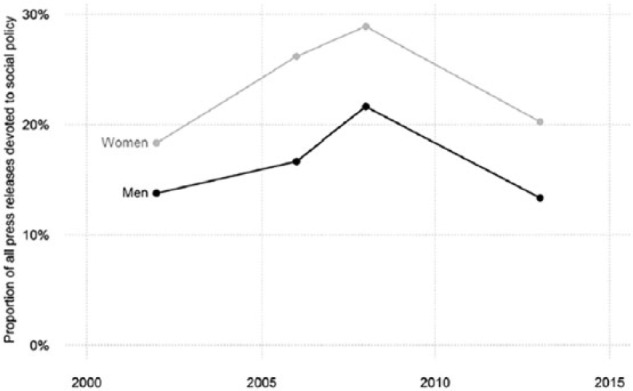
Proportion of all press releases devoted to social policy by gender. Dots represent the overall proportion of press releases on social policy issued by male (black) and female (grey) senders across four election campaigns (2002, 2006, 2008 and 2013).

Before moving to the multivariate models, a descriptive investigation of the two hypotheses will be presented. *H1* conjectures that women put greater emphasis on social policy than men. Figure 1 displays the proportion of all press releases dealing with social policy by gender and election. In all four election campaigns, there is a gender gap of at least five percentage points (p < 0.05 in all instances, two-sided test). Women thus address social policy at a consistently higher rate than men, providing initial support for the first hypothesis. The figure also shows some variation across campaigns. The peak in social policy emphasis in 2008 stems from the fact that the public debate centred on welfare measures to mitigate the effects of inflation and on proposals to change the early retirement scheme.

**Figure 2.**
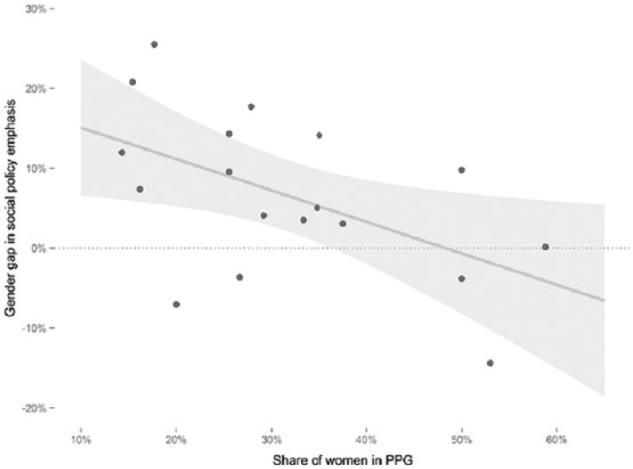
Gender balance and the gender gap in social policy emphasis. Gender gap in social policy emphasis is calculated by subtracting social policy emphasis among male senders from social policy emphasis among female senders. Regression line with 95 percent confidence intervals is shown. FPÖ 2006 dropped from the scatter plot due to a very low number of female senders (N = 6).

Next, Figure 2 inspects how the gender balance within parties (measured as the proportion of women in a party’s parliamentary delegation) influences the gender gap in social policy emphasis. The scatter plot in Figure 2 shows a very strong relationship (r = −0.61, p < 0.01, N = 19). Each dot represents one party-election observation. Parties with a very unequal gender distribution display a social policy gender gap of up to 25 percentage points, whereas parties with a more balanced gender makeup display much lower gender differences in their emphasis on welfare issues. Again, the descriptive evidence provides preliminary support for the second hypothesis.

To test whether the bivariate relationships hold in a multivariate context, two logistical regression models with random effects at the party-election level are specified. The dependent variable is binary and indicates whether the main issue of a press release (identified by coding title and subtitle) was in the area of social policy.

Model I tests the first hypothesis that women are more likely to focus on social policy issues. The coefficient for the female sender variable is positive and statistically significant (p < 0.001), its value of 0.266 translates into an odds ratio of 1.30, meaning that, all else being equal, the odds of social policy being the central concern of a press release increase by 30 percent when the sender is a woman. Considering that, in addition to the random effects, the regression model includes controls for ministers holding social policy portfolios, members of social policy committees in parliament, union officials, government participation, elite status and party ideology, this is quite a substantial effect.

**Figure 3.**
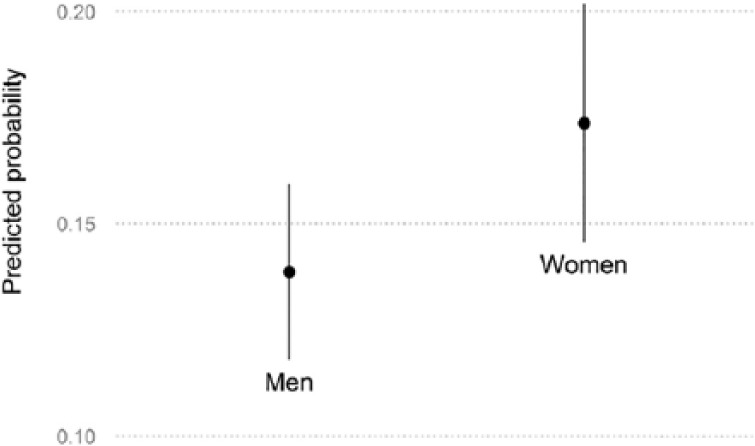
Predicted probability of emphasizing social policy by gender. Predicted probabilities calculated based on model I. All other covariates held constant at typical values (means for continuous variables and modes for categorical variables).

To illustrate the impact of gender, Figure 3 plots the predicted probabilities of emphasizing social policy for male and female senders. Holding everything else constant, the probability of social policy being the main issue of a press release increases from 13.9 percent to 17.4 percent between men and women (while the confidence intervals overlap, the difference between the two probabilities is significant at p < 0.05).

To test *H2*, model II in Table 3 introduces an interaction term between the female sender indicator and the share of women in the PPG. The interaction term is negative and significant (p < 0.05), indicating that the effect of gender on social policy emphasis declines as the proportion of women in a party’s parliamentary delegation increases. For each additional percentage point in the share of female MPs, the effect of gender on social policy emphasis (which, at 0.76, represents the hypothetical case of a party without women MPs) decreases by 0.015 (the coefficient for the interaction term).

**Table 3.** Binary logistic regression: explaining emphasis on social policy in campaign press releases.

	I	II
Female sender (*H1*)	0.266*** (0.0673)	0.744** (0.245)
Female sender × share of women in PPG (*H2*)		−0.0150* (0.00743)
Share of women in PPG	−0.0446*** (0.0109)	−0.0387*** (0.0111)
Minister of social, health or family affairs	0.948*** (0.263)	0.884*** (0.265)
Member of social, health or family affairs committee	0.804*** (0.0751)	0.792*** (0.0754)
Trade union officials	−0.228 (0.148)	−0.218 (0.147)
Member of government party	0.273* (0.121)	0.241* (0.119)
Member of political elite	−0.238*** (0.0676)	−0.229*** (0.0677)
Party ideology (left–right)	−0.198*** (0.0568)	−0.189*** (0.0558)
Intercept	0.601 (0.639)	0.412 (0.634)
$ln(\sigma_{u}^{2})$	−3.133*** (0.489)	−3.192*** (0.494)
N	7850	7850

PPG: parliamentary party group.

Cell entries are raw coefficients from binary logistic regression models with random effects at the party-election level; standard errors in parentheses; *p* *< 0.05, **p* *< 0.01, ***p* *< 0.001

**Figure 4.**
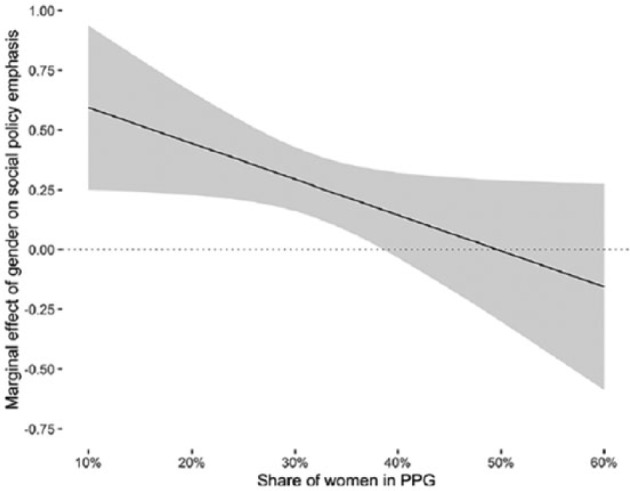
Marginal effect of gender on social policy emphasis by gender balance. Marginal effects plot based on model II. All other covariates held constant at typical values (means for continuous variables and modes for categorical variables).

Figure 4 illustrates this declining marginal effect across the empirical range of the gender balance variable. The effect is statistically significant for parties with low gender diversity and diminishes as the proportion of female MPs grows. The lower bound of the 95 percent confidence interval intersects the zero line at around 39 percent women MPs, meaning that above this threshold there is no statistically significant gender difference in the emphasis on social policy issues. Note that the effect depicted in Figure 4 is remarkably similar to the bivariate correlation displayed in Figure 2. A host of additional control variables has not affected the relationship much.

The results from the regression analysis thus yield solid support for *H2*. The data conform to the assumption that the gender gap in social policy emphasis is mediated by the gender diversity of a party. Women in male-dominated parties are much more likely to talk about questions of social policy than their male colleagues, whereas women and men in more gender-balanced parties address them at a similar rate.

A look at the remaining covariates in Table 3 reveals further statistically significant effects. First, the direct effect of gender balance on social policy emphasis (see model I) is negative. This is a reflection of the fact that the party with the highest average share of female MPs – the Greens – display the lowest overall saliency of social policy issues (see also Table 1). Despite usually promoting a generous welfare state (a winning position in Austrian politics), issue ownership considerations (i.e. the competence advantage that the Social Democrats enjoy among Austrian voters) appear to have kept the party from making social policy a central feature of its campaign agenda. Yet, it is important not to confuse two things: While the gender gap in social policy emphasis is similar (i.e. small) in the two left-of-centre parties, SPÖ politicians display a greater overall emphasis on social policy than their Green colleagues.

Furthermore, politicians who are institutionally linked to social policy areas (as ministers or members of a parliamentary committee) have considerably higher propensities to talk about these issues. The coefficients for the minister and committee member variables in the two models translate to odds ratios between 2.2 and 2.6, suggesting that these politicians are more than twice as likely to address issues of welfare, health, family or childcare in their press releases. Trade union members, by contrast, do not talk about social policy more often than other politicians.

Similarly, politicians from government parties display a 30 percent higher propensity to address social policy matters than their opposition counterparts. Note that – unusually for Austria – there was quite some alteration in the partisan makeup of government during the period of observation. Centre-right cabinets were in office before the 2002 (ÖVP–FPÖ) and 2006 (ÖVP–BZÖ) elections, after which a grand coalition of SPÖ and ÖVP was reinstated (2008 and 2013).

Interestingly, however, elite politicians are almost 20 percent less likely to address social policy questions than their rank-and-file peers. Closer inspection of the data reveals that most substantive policy areas are underrepresented in the press releases of party elites. These high-ranking politicians have a higher probability of addressing non-policy issues, such as scandals, political performance and efficiency, or attacking their opponents.

Finally, the party ideology predictor also comes out significant. However, this is largely an artefact of this variable’s strong correlation with the share of female MPs (left parties have considerably more). Excluding the gender balance variable renders the ideology predictor insignificant (note, however, that the reverse is not true).^7^

## Discussion and conclusion

This study has provided strong evidence for the gendered nature of social policy as a political issue. It represents one of the most comprehensive analyses of the link between welfare issues and gender in Europe to date. The results reveal that women are considerably more likely to address questions, such as pensions, health, family, childcare or poverty, in election campaigns than men. This is consistent with findings from the literature on legislative behaviour and organization, portfolio allocation and campaign communication.

The novel contribution that this article makes to the literature on social policy and gender, however, is to identify gender balance at the group level as an important mediator of this relationship. The effect of gender at the individual level is hypothesized to vary with the intra-party gender distribution. As the descriptive evidence and the statistical models demonstrate, the gender gap in social policy emphasis indeed shrinks towards zero as the gender composition in a party’s parliamentary delegation (or, alternatively, the gender composition of party-election lists, see the online appendix) reaches parity.

This result supports the argument that diversity at the group level influences the degree of role differentiation and functional segregation along gender lines at the individual level. The quantitative parity between men and women politicians thus has an impact on the qualitative division of labour within political parties. As a consequence, social policy questions lose their designation as women’s issues.

To be sure, a considerable degree of gender segregation is baked into some of the control variables. For instance, almost half of all seats on the parliamentary committees included in the analysis (social affairs, family and health) are occupied by women, even though the overall proportion of female legislators has never been more than a third during the period of observation. This means that, even in gender-equal parties, women will likely address social policy matters more often, because they are more likely to sit on the respective committees. The gender–social policy link is thus not just a matter of campaign communication. Rather it is a phenomenon deeply entrenched into the distribution of positions between men and women within the most important political institutions. While reaching numerical parity certainly constitutes a step in the right direction, it may not be enough to overcome the functional segregation of men and women in politics – as the proponents of tokenism theory have repeatedly suggested (Yoder, 1991, 1994).

As with all single-country studies, there are some caveats to how far we should generalize from the findings. While Austria may be considered representative of other West European democracies in many respects, it should be noted that overall gender norms are still more traditional than, for instance, in many Northern European countries.

Female political representation is still well below gender parity, and women are especially scarce in party leadership positions (Ennser-Jedenastik and Müller, 2014). Also, public opinion and many welfare arrangements still embrace the male breadwinner model (Budig et al., 2012), giving Austria one of the largest gender pay gaps in Europe (Böheim et al., 2013). What is more, opinion about gender roles is more polarized in Austria than in most other European societies, and much of that polarization is along party political lines (Hudde, 2016).

It could therefore be argued that the patterns found in the analysis are likely to be less pronounced in countries with more egalitarian norms and a greater consensus on gender roles. Comparative research may help clarify in the future to what extent the results presented here are replicated in other political systems.

## Supplementary Material

Supplementary material

## Notes

1. Dolan’s study differs, in that it looks at campaign websites rather than political ads. Websites present fewer constraints for candidates in terms of issue focus and prioritization, which may explain the non-finding.

2. In some cases, there may be a purely quantitative effect. As the recruitment pool for an office becomes more female, the probability of a woman being appointed increases. However, this does not explain why women are disproportionately recruited to certain position such as those dealing with social policy.

3. It should be noted that a minority of press releases (790 out of the total of 8640) do not identify any individual politician as sender but just feature the party as the messenger. Since gender cannot be coded for these observations, they have been excluded from the analysis.

4. While there are certainly a large number of categories, the specificity actually helps coders to find the best-matching issue. Since resource constraints dictated that each press release was only coded once, inter-coder agreement was assessed with a random sample of 100 press releases that was given to six independent coders. They identified social policy issues with a very high degree of reliability (Krippendorff’s α = 0.86).

5. The minority of press releases featuring two senders (6%) is coded 1 on this variable when at least one sender is female.

6. A reasonable alternative would be to use the proportion of women among party candidates for each election. This variable is strongly correlated with PPG shares (r = 0.86 for 20 party-election observations, p < 0.001) and yields virtually identical results in the multivariate analysis, see Table A1 in the online appendix.

7. Also, including an interaction of ideology with gender does not alter the substantive conclusions regarding *H1* and *H2*.
