# Supplementary material for: Campaigning on the welfare state: The impact of gender and gender diversity
Source: J Eur Soc Policy. 2017 Feb 1;27(3):215–28. doi: 10.1177/0958928716685687 (PMC5505229; doi:10.1177/0958928716685687)
Supplement: Supplementary material [file Online_appendix.pdf]

O N L I N E   A P P E N D I X

Campaigning on the welfare state: The impact of gender and gender diversity

Laurenz Ennser-Jedenastik
University of Vienna
Department of Government
laurenz.ennser@univie.ac.at

Table A1 Alternative specification of models using share of women candidates

	III	IV
Female sender (H1)	0.262***<br>(0.0672)	1.032**<br>(0.320)
Female sender × share of women in PPG (H2)		-0.0186*<br>(0.00758)
Share of women candidates on election list	-0.0236<br>(0.0216)	-0.0200<br>(0.0214)
Minister of social, health, or family affairs	0.938***<br>(0.264)	0.870**<br>(0.266)
Member of social, health, or family affairs committee	0.805***<br>(0.0753)	0.804***<br>(0.0753)
Trade union official	-0.214<br>(0.148)	-0.202<br>(0.148)
Member of government party	0.408*<br>(0.207)	0.386#<br>(0.205)
Member of political elite	-0.225***<br>(0.0677)	-0.210**<br>(0.0680)
Party ideology (left–right)	-0.0883<br>(0.105)	-0.0914<br>(0.103)
Intercept	-0.563<br>(1.341)	-0.668<br>(1.326)
$\ln(\sigma_u^2)$	-2.350***<br>(0.412)	-2.379***<br>(0.414)
N	7,850	7,850

Note: Cell entries are raw coefficients from binary logistic regression models with random effects at the party-election level; standard errors in parentheses; * $p < 0.05$, ** $p < 0.01$, *** $p < 0.001$

Table A2 Alternative specification of models using fixed effects

	V	VI
Female sender (H1)	0.266***	0.690**
	(0.0674)	(0.251)
Female sender × share of women in PPG (H2)		-0.0133#
		(0.00759)
Minister of social, health, or family affairs	0.964***	0.906***
	(0.264)	(0.266)
Member of social, health, or family affairs committee	0.796***	0.786***
	(0.0755)	(0.0758)
Trade union official	-0.237	-0.228
	(0.148)	(0.148)
Elite politician	-0.238***	-0.230***
	(0.0679)	(0.0681)
N	-0.238***	-0.230***
	(0.0679)	(0.0681)

Note: Cell entries are raw coefficients from binary logistic regression models with fixed effects at the party-election level; note that all covariates that vary only between parties and elections are dropped from the estimation; standard errors in parentheses; # $p < 0.1$, * $p < 0.05$, ** $p < 0.01$, *** $p < 0.001$

Table A3 Policy areas included in dependent variable

Issue
Social policy, welfare state (general)
Social justice, redistribution
Social insurance contributions
Poverty reduction, basic income
Social housing, housing subsidies
Support for unemployed, unemployment administration
Health care, health care system, health insurance
Care for the elderly, care for disabled people, financing of care
Pensions, pensioners
Families, children, youth, family benefits, child benefits
Child care, kindergarten, early childhood education
Welfare provisions for immigrants
